# Spatial features of tumor-infiltrating lymphocytes in primary lesions of lung adenocarcinoma predict lymph node metastasis

**DOI:** 10.1186/s12967-025-06860-1

**Published:** 2025-07-25

**Authors:** Huibo Zhang, Ming Luo, Junwei Feng, Juan Tan, Yan Jiang, Dmitrij Frishman, Yang Liu

**Affiliations:** 1 Department of Bioinformatics, TUM School of Life Sciences, Technical University of Munich, Freising, Germany; 2 Cancer Center, Renmin Hospital of Wuhan University, Wuhan, China; 3 Department of Oncology, Taihe Hospital, Hubei University of Medicine, Hubei, China; 4 Department of Interventional Radiology, Renmin Hospital of Wuhan University, Wuhan, China; 5 Department of Pathology, The Third Xiangya Hospital, Central South University, Changsha, China

**Keywords:** Tumor-infiltrating lymphocytes, TILScout, Whole slide images, Lung adenocarcinoma, Lymph node metastasis

## Abstract

**Background:**

Lymph node metastasis (LNM) is critical for staging, prognosis, and treatment decisions in lung adenocarcinoma (LUAD). While tumor‐infiltrating lymphocytes (TILs) have demonstrated prognostic value, their role in LNM risk remains uninvestigated. This study evaluates the relationship between TIL features from primary tumor whole slide images (WSIs) and LNM in LUAD.

**Methods:**

TILScout was utilized to derive patch-level TIL scores and generate global TIL maps from primary tumor WSIs. Hot spot analysis and deep learning-based feature extraction followed by K-means clustering were applied to identify and characterize spatial TIL clusters (sTILCs) from the global TIL maps. Random forest models incorporating clinical/pathological data with (M1) and without (M2) TIL features (TIL scores and sTILCs) were developed on a training cohort (N = 312) to predict LNM, and performance was compared across validation (N = 78) and independent test cohorts (N = 148).

**Results:**

Two sTILC types (“TIL-cold” cluster [sTILC1] and “TIL-hot” cluster [sTILC2]) were identified. Model M1 significantly improved LNM prediction over M2, with AUCs increasing from 0.63 to 0.78 (Z = 5.366, P < 0.001) and from 0.61 to 0.72 (Z = 1.999, P = 0.046) in the training and validation cohorts, and from 0.69 to 0.80 (Z = 3.030, P = 0.002) in the test cohort. Decision curve analysis indicated that M1 provided greater net benefit across a broad spectrum of threshold probabilities. Importantly, patients with lower TIL scores and/or classified as sTILC1 consistently had an increased risk of LNM.

**Conclusions:**

Spatial TIL features in primary tumors are linked to LNM in LUAD, thereby enabling the identification of high-risk patients and guiding personalized treatment strategies.

**Supplementary Information:**

The online version contains supplementary material available at 10.1186/s12967-025-06860-1.

## Introduction

Lung cancer remains the leading cause of cancer-related morbidity and mortality globally, accounting for approximately 12.4% of all cancer diagnoses and 18.7% of cancer deaths worldwide [1]. Non-small cell lung cancer (NSCLC) represents about 90% of lung cancer cases, with lung adenocarcinoma (LUAD) being the most common subtype [2]. The presence of lymph node metastasis (LNM) is critical in the staging, prognosis, and management of lung cancer, influencing both the likelihood of recurrence following surgical intervention and subsequent treatment strategies as per the National Comprehensive Cancer Network (NCCN) guidelines (www.nccn.org/guidelines/) [3–6]. Moreover, LUAD is particularly associated with a higher incidence of occult nodal metastasis compared to other NSCLC subtypes [7], with smaller lymph nodes in hilar or mediastinal stations frequently harboring metastatic disease [8]. Thus, identifying factors that influence LNM is crucial for accurate diagnosis and effective treatment planning, especially for patients eligible for curative-intent therapy.

In the evolving landscape of cancer immunotherapy, the prognostic significance of tumor-infiltrating lymphocytes (TILs) is garnering attention. Despite ongoing debates regarding their role across various cancers [9], recent evidence underscores a positive correlation between TIL density and improved prognosis in LUAD in the context of immunotherapy [10–15]. Moreover, spatial patterns of TILs have been linked to recurrence risk in early-stage NSCLC [16], suggesting a complex interplay between the tumor immune microenvironment and cancer progression.

While a number of studies have explored the clinicopathological factors, such as age and tumor size [17], which were identified as the independent risk factors for LNM, the impact of TIL features on LNM risk remains poorly understood. Existing studies are either limited by small sample sizes or lack a focus on the immunological aspects within the tumor microenvironment [18, 19]. Therefore, further research into whether TIL features could influence LNM in LUAD patients is imperative.

We have previously developed a pan-cancer applicable approach named TILScout [20] for calculating TIL scores and generating global TIL maps based on whole slide images (WSIs). In this study, we sought to evaluate the association between LNM and the TIL features derived from primary tumor WSIs of LUAD patients. Our goal was to enhance the current understanding of the immunological underpinnings of tumor progression and to aid in refining prognostic models and therapeutic approaches for LUAD, potentially leading to more personalized and effective cancer treatment strategies.

## Materials and methods

### Study cohorts

In this work data from two LUAD cohorts were used: the primary cohort from the Cancer Genome Atlas (TCGA) database (TCGA cohort) and the test cohort from The Third Xiangya Hospital, Central South University (XY cohort). The retrospective study on the XY cohort received approval from the institutional committee and informed consent was waived. For the TCGA cohort, clinical and pathological information such as age, and pathological T and N stages, along with TPM-normalized RNA-seq data and Hematoxylin and Eosin (H&E)-stained whole slide images (WSIs) were obtained via the Genomic Data Commons (GDC) portal (https://portal.gdc.cancer.gov/repository). Cases with unknown stage or age and WSIs that featured artificial markings, overlapping tissues, or bubbles were excluded from consideration. For the XY cohort, we randomly selected 150 high-quality H&E-stained pathological slides of primary tumors from surgically resected LUAD cases diagnosed between January 2020 and June 2023. These slides were scanned at 20 × magnification and images were saved in *svs* format. Clinical information for cancer patients was collated from electronic medical records. Two cases in the XY cohort were excluded due to the absence of pathological T stage and duplicate slides from the same case, respectively. The final dataset included 390 cases from the TCGA cohort and 148 cases from the XY cohort. LNM status was categorized into two groups: pathological N0 stage, indicating no LNM, and any of the N1, N2, or N3 stages, indicating the presence of LNM.

### TIL scores and global TIL maps of WSIs derived from TILScout

We previously developed a pan-cancer applicable method named TILScout [20], which comprises two components: an InceptionResNetV2-based classifier for patch label prediction and a software package for automated computation of TIL scores and generation of global TIL maps from WSIs. The classifier was trained on a manually labeled dataset containing 90,488 patches from 28 cancer types, categorized into three labels: TIL-positive, TIL-negative, and necrotic/other.

Each WSI at 20 × magnification was automatically divided into 150 × 150-pixel non-overlapping patches utilizing the OpenSlide library in Python. To eliminate the effect of color variations in H&E-stained images from different institutions, patches were standardized using Macenko's color normalization method [21]. Predictions were made for each patch within a WSI. Subsequently, the corresponding TIL score was derived, and the global TIL map was constructed for each WSI. A TIL score for a WSI is derived by dividing the number of predicted TIL-positive patches by the total number of non-necrotic/other patches. The global TIL map illustrates the distribution of TIL-positive patches across the entire WSI. In cases involving multiple WSIs for a given patient, the TIL score represents the mean of scores from all slides.

### Hot spot analysis for TIL maps

Hot spot analysis is a mapping technique used to detect spatial clusters by examining the distances between different elements. This approach was employed to identify areas within a geographical region where values (e.g., specific biomarker levels, event frequencies, or other quantifiable phenomena) significantly deviate from the norm, being either higher or lower than anticipated [22]. By calculating the Getis-Ord Gi* statistic, hot spot analysis evaluates local spatial autocorrelation [23], revealing clusters of high values (hot spots) (e.g., high biomarker concentrations or event frequencies) and low values (cold spots). Based on this inspiration, we performed hot spot analysis of TIL maps to explore spatial distribution characteristics of TILs. Each patch within a TIL map was treated as a distinct ‘geographic’ spot. TIL-positive patches were assigned a label of 1, while all others received a label of 0. Utilizing the ESDA library in Python, we computed local Gi* statistics for each patch. These statistics indicate whether a certain WSI area is significantly enriched or depleted in positive TIL patches compared to its neighboring areas. Hot spot maps were then generated based on the ‘geographic’ locations of the patches and their corresponding Gi* statistics, effectively visualizing areas of significant TIL presence.

### Spatial TIL features derived from hot spot maps

A deep learning encoder architecture was employed to extract spatial features from hot spot maps. Hot spot maps, treated as input images, were uniformly resized to dimensions of 1024*1024 pixels, incorporating three color channels (RGB). These images were subsequently processed through a sequence of four convolutional layers. Each convolutional layer was followed by a max pooling operation, which progressively reduced the spatial dimensions of the feature maps from 1024 * 1024 * 3 to 16,384-dimensional feature vectors. To focus on the most informative features, the high-dimensional data were then subjected to principal component analysis (PCA), which resulted in condensing the features into 244 principal components per image. Furthermore, the K-means method was utilized to obtain spatial clusters of TILs (sTILCs) for each sample. The optimal number of clusters was determined using a consensus voting method among three established clustering evaluation metrics—the Silhouette Score [24], Davies-Bouldin Index [25], and Calinski-Harabasz Index [26]- as implemented in the scikit-learn Python library. Each method provides a different perspective on cluster cohesion and separation, ensuring a comprehensive evaluation of clustering effectiveness. Notably, a consistent clustering strategy was employed across both the TCGA and XY cohorts to ensure the uniformity of the analysis.

### LNM prediction model

The TCGA cohort was randomly divided into a training cohort, and a validation cohort at a ratio of 8:2. Random forest models were developed to predict LNM in the training cohort and were validated in the validation cohort. Cross-validation was used to optimize model hyperparameters during model training. Models integrating both clinical/pathological data (age, T stage) and TIL features (TIL scores and sTILCs) (model M1, TIL feature-based model) were compared with models relying solely on clinical/pathological data (model M2). The performance of the models was subsequently evaluated in the XY cohort.

We also investigated the potential impact of incorporating different features into model M1. Specifically, we explored the performance of model M1 under two additional scenarios: (1) including all 244 PCA-derived features in place of the sTILCs and (2) using only the two most important principal components (PC1 and PC2) instead of the sTILCs. These variations of model M1 were then compared to the original model M1 that included the sTILCs, providing further insights into the most effective way to represent TIL spatial characteristics for predicting LNM.

### Biological relevance of TIL features

Correlation analyses between TIL features and putative proportions of different immune cell types established based on gene expression data by the CIBERSORT algorithm [27] were conducted. TIL score-related biological functions were evaluated using Gene Ontology (GO) and Kyoto Encyclopedia of Genes and Genomes (KEGG) analyses through the R package clusterProfiler [28], based on the top 1,000 genes most correlated with TIL scores in the TCGA cohort. Further Gene Set Enrichment Analysis (GSEA) was performed on the ranked list of 1,000 genes for two types of gene annotations— Reactome [29] pathways and hallmark gene set [30]—using ReactomePA [31] and clusterProfiler R packages, respectively. Annotations with a normalized enrichment score (NES) > 2 and an adjusted P value < 0.05 were deemed significantly enriched.

### Statistical analysis

Categorical variables were analyzed using the Chi-square test, while continuous variables were compared using the t-test. Model discrimination was assessed via receiver operating characteristic (ROC) curves and the area under the ROC curve (AUC) in the training, validation, and test cohorts. The Brier score and Delong tests [32] were used to evaluate the performance of models. A lower Brier score indicates better model performance. The z-score calculated by the Delong test assesses the improvement in AUCs of different models, while the p-value below 0.05 derived from the z-score indicates statistically significant difference of AUC values between the models, suggesting that one model has significantly better discriminatory power than the other. Furthermore, SHAP (SHapley Additive exPlanations) [33] values for each variable and instance in the M1 model were calculated, elucidating the contribution of each feature to the prediction of LNM.

Decision curve analysis (DCA) [34, 35] (www.decisioncurveanalysis.org) was used to analyze and compare the net benefit of different models, and test the clinical usefulness of the TIL feature-based model in predicting LNM. The net benefit is conceptualized as a quantitative metric that assesses the utility of employing a specific diagnostic or predictive model across various decision thresholds, effectively balancing the trade-offs between false positives and true positives.

All statistical analyses mentioned above were conducted using R (version 4.3.1) and Python (version 3.9.3) languages. A P-value of < 0.05 was considered statistically significant.

## Results

### Overview of the study

**Fig. 1:**
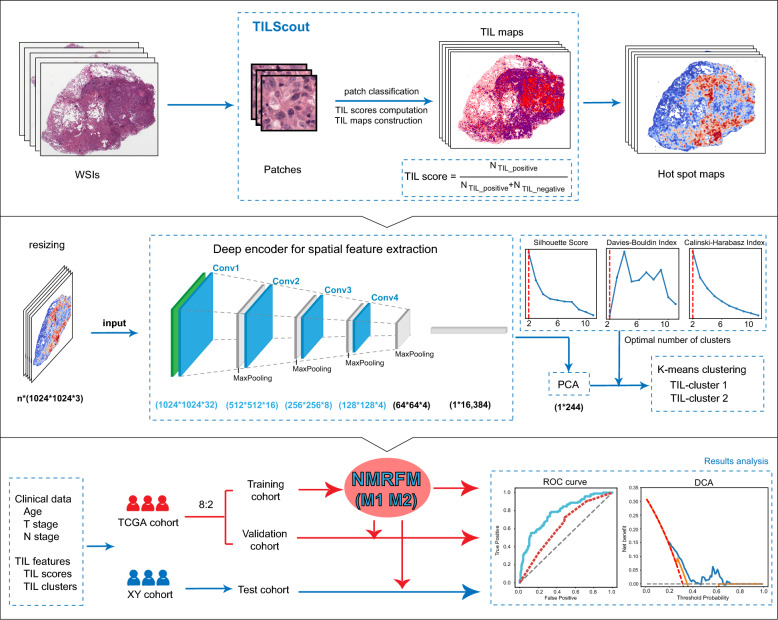
Computational pipeline for predicting LNM. In the first step, TILScout was utilized to calculate TIL scores and generate TIL maps from WSIs. Hot spot maps were derived from the TIL maps, highlighting regions with high density of TIL infiltration. In the second step, these hot spot maps were further processed to extract spatial TIL clusters using a deep encoder architecture after being resized to dimensions of 1024 × 1024 pixels. The encoder consists of four convolutional layers, each followed by max-pooling operations, progressively down-sampling the spatial dimensions and producing a 16,384-dimensional feature vector per image. Next, Principal Component Analysis (PCA) was applied to further reduce the feature space to 244 principal components per image. K-means clustering was performed to identify two spatial clusters based on consensus voting across three clustering evaluation metrics: Silhouette Score, Davies-Bouldin Index, and Calinski-Harabasz Index. In the final step, Node Metastasis Random Forest Models (NMRFMs) were trained on the training cohort and validated and tested on separate validation and test cohorts. Two models were developed: Model M1, incorporating clinical/pathological data (age, T stage) and TIL features (TIL scores and spatial TIL clusters), and Model M2, trained solely on clinical/pathological data (age, T stage). The performance of both models was evaluated and compared across cohorts

The objective of this study was to investigate the relevance of TIL distribution in primary tumor tissues for predicting LNM in LUAD (Fig. 1). Patch-level TIL scores and global TIL maps were derived from whole slide images (WSIs) using our previously developed method TILScout [20]. Two types of spatial TIL clusters (sTILCs)—(“TIL-cold” cluster (sTILC1) and “TIL-hot” cluster (sTILC2))—representing the spatial features of TILs, were derived by the K-means method from TIL hot spot maps. The performance of random forest models for predicting LNM, developed in the training cohort, was evaluated by integrating clinical/pathological data with and without patch-level TIL features (TIL scores and sTILCs). These models were systematically compared across the validation and independent test cohorts. This comprehensive approach guarantees a robust evaluation of the predictive power of TIL features for the likelihood of LNM and their clinical benefits.

### Derivation of TIL features

**Fig. 2:**
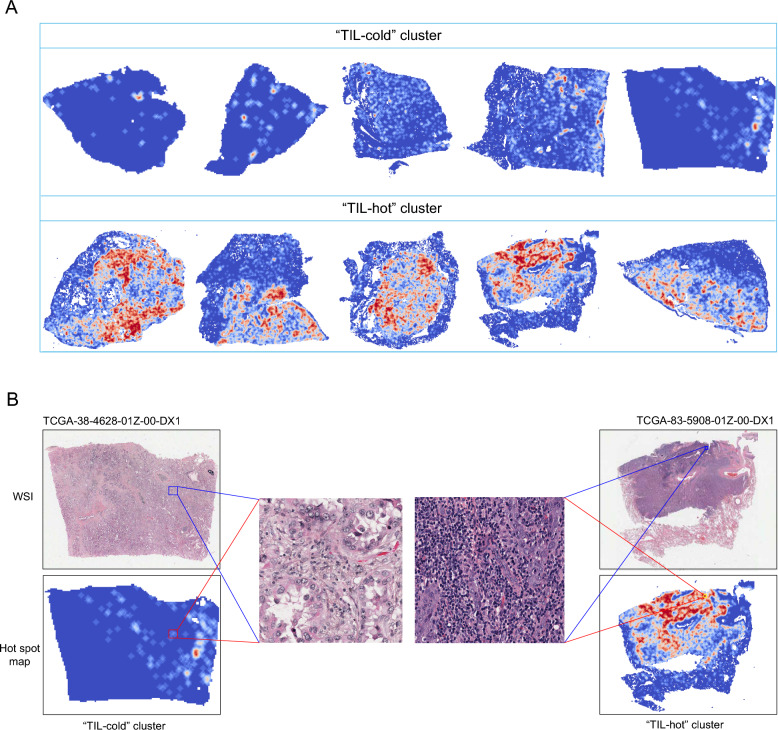
Hot spot maps and spatial TIL clusters. **A** Examples of hot spot maps. A gradient color scale from blue to red represents varying TIL densities. Red areas highlight regions of high TIL density. **B** Two examples of WSIs with the corresponding hot spot maps and sTILCs. Two clusters were identified: the “TIL-cold” cluster, characterized by sparse TIL density across hot spot maps; and the “TIL-hot” cluster, defined by densely concentrated TIL distribution

A TIL score and a TIL map were automatically derived for each sample/WSI by TILScout. Hot spot maps were generated based on the locations of patches and their corresponding Gi* statistics. In Fig. 2A, we provide some examples depicting the TIL hot spot maps. A gradient from blue to red signifies varying TIL densities, with red areas indicating regions of high density. This visualization effectively underscores the heterogeneity of TIL distribution, facilitating the extraction of spatial TIL features from WSIs.

Each resized hotspot map was processed by a deep learning encoder, followed by PCA, which reduced the spatial dimensions from 1024 * 1024 * 3 to 244 principal components (PCs). All hot spot maps were subsequently clustered into two distinct groups using the K-means method based on these 244 PCs, with the optimal number of clusters determined to be 2 based on the Silhouette Score, Davies-Bouldin Index, and Calinski-Harabasz Index (Supplementary Fig. 1). The dimensionality reduction and clustering methods were consistently applied to both the TCGA and XY cohorts to ensure identical processing across the entire analytical workflow.

To better understand the intrinsic differences between the two clusters, we analyzed the distinctive characteristics observed in the hot spot maps for each group. We assigned descriptive labels to the spatial TIL clusters (sTILCs): the first group, characterized by sparse TIL density within hot spot maps, was termed the"TIL-cold"cluster (sTILC1), whereas the second group, featuring high TIL density, was labeled the"TIL-hot"cluster (sTILC2), (Fig. 2A-B). These labels effectively capture the spatial heterogeneity in TIL distribution between the clusters, enabling a more interpretable analysis of TIL spatial patterns across samples.

### Baseline characteristics of the patient cohorts

**Fig. 3:**
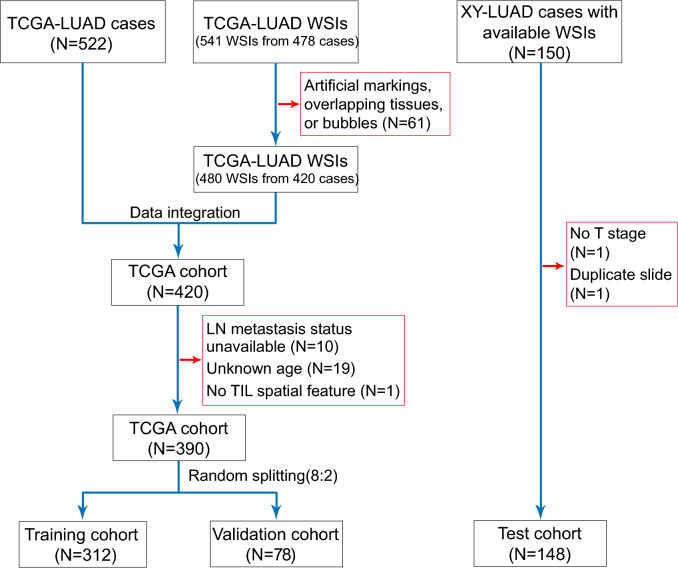
Process of the patient enrollment for the training, validation, and test cohorts

The data enrollment process regarding the training, validation, and test cohorts is illustrated in Fig. 3. The baseline characteristics of the three cohorts are summarized in Table 1, including age, pathological T and N stage, TIL scores, and spatial TIL clusters. Notably, a significant difference was observed in the case distribution of the T stage between the groups with and without LNM. TIL scores decreased in the LNM group across the three cohorts, with this trend reaching statistical significance in the test cohort. Regarding sTILCs, there was no significant difference in the case distribution among the three cohorts (Table 1, P* and P** value > 0.05). Additionally, no differences were observed in the variable distributions between the training and validation cohorts. However, significant differences were noted in the distributions of variables such as age, stage, and TIL scores (P** value < 0.001) between the test and the training cohorts.

**Table 1:** Characteristics of patients in the training, validation, and XY cohorts

Characteristics		Training cohort (N = 312)				Validation cohort (N = 78)				P*	Test cohort (N = 148)				P**
LN status		Total	Metastasis	Non-metastasis	p		Metastasis	Non-metastasis	p			Metastasis	Non-metastasis	p	
Case number (N)		312	98	214	–	78	25	53	–		148	76	72	–	
Age (N)	< 45	13	4	9	0.490	4	1	3	0.737	0.288	7	3	4	0.874	0.003
	45–65	147	51	96		39	18	21		–	94	48	46		
	> 65	152	43	109		35	6	29		–	47	25	22		
T stage (N)	T1	118	24	94	0.007	27	3	24	0.028	0.895	96	33	63	< 0.001	< 0.001
	T2	157	61	96	–	41	17	24	–	–	40	34	6	–	–
	T3	29	9	20	–	7	3	4	–	–	10	7	3	–	–
	T4	8	4	4	–	3	2	1	–	–	2	2	0	–	–
TIL scores (average)		0.260	0.239	0.270	0.172	0.252	0.200	0.277	0.021	0.713	0.347	0.289	0.409	< 0.001	< 0.001
TIL spatial features (N)	sTILC1	150	52	98	0.284	45	18	27	0.131	0.164	70	46	24	0.002	0.955
	sTILC2	162	46	116	–	33	7	26	–	–	78	30	48	–	

The P* value is for comparing the training cohort with the validation cohort; the P** value is for comparing the training cohort with the test cohort. Categorical variables were analyzed using the Chi-square test, while continuous variables were compared using the t-test

### Development, validation, and testing of the prediction models

**Fig. 4:**
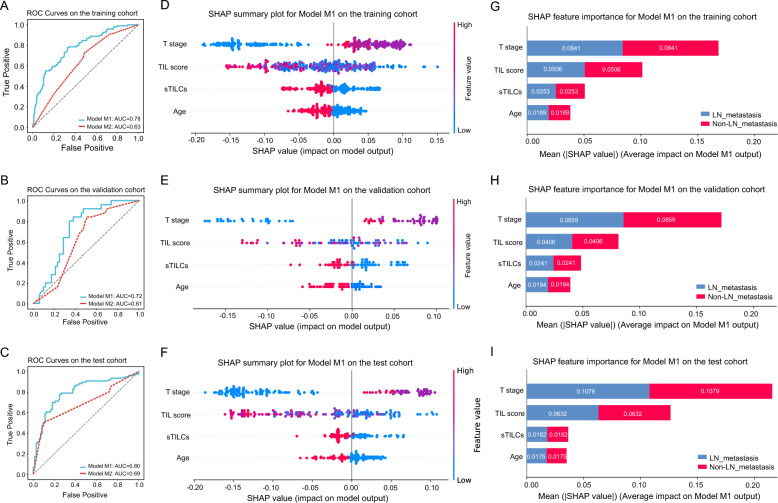
Performance of two models and feature impact for model M1 derived by SHAP. Figure 4A-C, ROC curves, illustrating the performance of the two models on the training, validation, and test cohorts with the values. Figure 4D-F, SHAP summary plots, highlighting the contribution of 4 features to the predictions of model M1 across the training, validation, and test cohorts. SHAP values were calculated based on the trained model; positive values represent a favorable impact on the prediction, while negative values indicate a contrary effect. Variables were assigned by values (feature values): Age, 0: < 45, 1: 45–65, 2: > 65; T stage, 0: T1, 1: T2, 2: T3, 3: T4; sTILCs, 1: “TIL-cold” cluster, 2: “TIL-hot” cluster. Figure 4G-I, SHAP feature importance plots, displaying the mean absolute SHAP value for each feature and ranking the features by their average impact on the output of model M1 across the cohorts

Two random forest models were developed using the training cohort: i) model M1, which integrates clinical/pathological features with TIL features (TIL scores, sTILCs), and ii) model M2, solely based on clinical/pathological features. The M1 model exhibited higher AUC values across all three cohorts compared to the M2 model (training cohort: 0.78 vs. 0.63; validation cohort: 0.72 vs. 0.61; test cohort: 0.80 vs. 0.69) (Fig. 4A-C). Based on the DeLong test, the M1 model significantly outperformed the M2 model in terms of AUC across the three cohorts with very high z-scores across the training (5.366, P < 0.001), validation (1.999, P = 0.046), and test cohorts (3.030, P = 0.002). Brier scores in the training (M1: 0.200 vs. M2: 0.233), validation (M1: 0.215 vs. M2: 0.228), and test (M1: 0.206 vs. M2: 0.227) cohorts indicated that the M1 model had higher accuracy and lower error in probability predictions (Table 2).

**Table 2:** Comparison between M1 and M2 models by Delong test and Brier score

Dataset	Delong test (Model M1 vs. Model M2)			Brier Score	
	Z score	P value		Model M1	Model M2
Training cohort	5.366	< 0.001		0.200	0.233
Validation cohort	1.999	0.046		0.215	0.228
Test cohort	3.030	0.002		0.206	0.227

Besides, the model M1 in the first scenario (using all 244 PCA-derived features instead of sTILCs) showed clear overfitting on the training set (AUC = 1.00) (Supplementary Fig. 2 A). This was further evidenced by the poor AUC value on the validation (AUC = 0.70) and independent test set (AUC = 0.57) (Supplementary Fig. 2B-C), which was even inferior to that of model M2 (AUC = 0.69). Similarly, model M1 in the second scenario (using PC1 and PC2 instead of sTILCs) also exhibited a markedly higher AUC value in the training set than in the validation and independent test sets (0.89 vs. 0.63 vs. 0.78) (Supplementary Fig. 2D-F), indicating a potential risk of overfitting. These findings suggest that incorporating all extracted PCA features or the top two PCs may not provide a robust representation of TIL features for predicting LNM compared with the sTILCs.

We utilized SHAP summary plots to visualize the relationships between feature values and their impacts on the prediction of the M1 model (Fig. 4). Higher T stage was associated with increased predicted risk of LNM (positive SHAP values). In contrast, higher TIL scores and the “TIL-hot” cluster (sTILC2) pointed to reduced predicted risk (negative SHAP values) in the training cohort (Fig. 4D). The same trends persisted in the validation and test cohorts (Fig. 4E-F). Additionally, the SHAP feature importance for the M1 model was established by averaging absolute SHAP values per feature (Fig. 4G–I). The top three variables of feature importance in the M1 model were T stage, TIL scores, and sTILCs in the training, validation, and test cohorts. T stage was the most critical feature, with an average absolute SHAP value of 0.0841 in the training cohort, 0.0859 in the validation cohort, and 0.1079 in the test cohort, indicating its significant contribution to the predicted risk of LNM. TIL scores contributed with an average absolute SHAP value of 0.0506 in the training cohort, 0.0406 in the validation cohort, and 0.0632 in the test cohort. sTILCs contributed with an average absolute SHAP value of 0.0253, 0.0241, and 0.0182 in the training, validation, and test cohorts, respectively. These absolute SHAP values represent the average magnitude of impact each feature has on the model's predictions, highlighting their relative importance in predicting LNM.

**Fig. 5:**
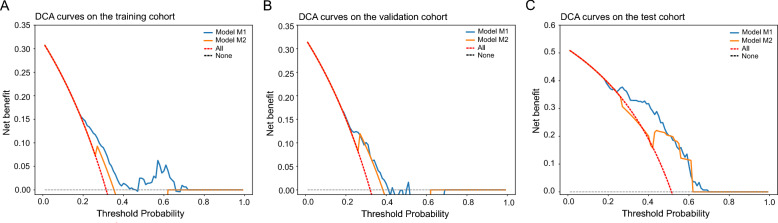
Decision Curve Analysis (DCA) comparing models M1 and M2 across the training, validation, and test cohorts. The DCA plots indicate that model M1 demonstrates a consistently higher net benefit compared to model M2, particularly within the threshold probability range of 0.2 to 0.7 in the training cohort, 0.2 to 0.5 in the validation cohort, and 0.2 to 0.7 in the test cohort. Threshold probability is the minimum probability of a positive outcome at which a patient would opt for an intervention

Decision curve analysis (DCA) revealed the comparative net benefits of models M1 and M2, as illustrated in Fig. 5. Across a broad spectrum of threshold probabilities within the training, validation, and test cohorts, model M1 consistently demonstrated better net benefit compared to model M2. This indicates that model M1 is notably more effective in predicting LNM.

### Biological relevance of TIL scores

**Fig. 6:**
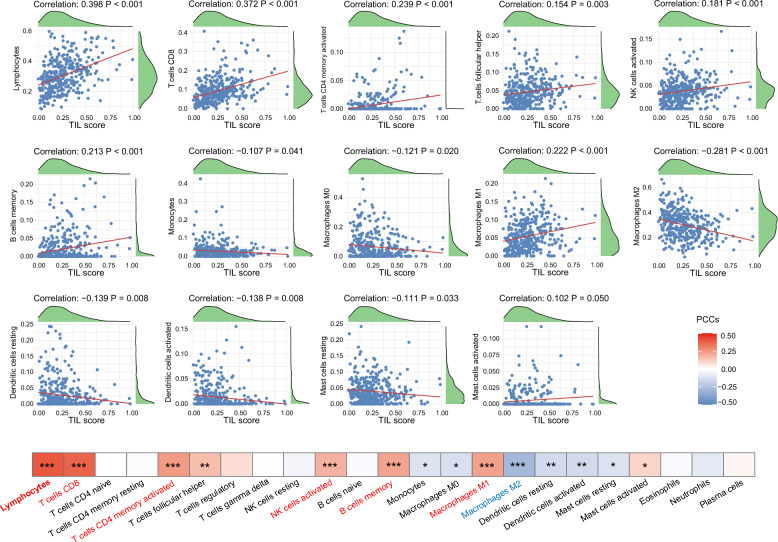
Correlation between TIL scores and immune cell infiltration. PCCs, Pearson correlation coefficients. “*”, P < 0.05; “**”, P < 0.01; “***”, P < 0.001

The correlation analysis between TIL scores and putative immune cell fractions revealed that TIL scores exhibited a highly significant positive correlation with lymphocyte infiltration (correlation coefficient: 0.398, P < 0.001), primarily due to the contributions of CD8 + T cells (correlation coefficient: 0.372, P < 0.001), CD4 + memory activated T cells (correlation coefficient: 0.239, P < 0.001), activated NK cells (correlation coefficient: 0.181, P < 0.001), and memory B cells (correlation coefficient: 0.213, P < 0.001) (Fig. 6). Additionally, TIL scores were highly positively correlated with M1 macrophage infiltration (correlation coefficient: 0.222, P < 0.001), which primarily promotes inflammation and anti-tumor activity, and were negatively correlated with M2 macrophage infiltration (correlation coefficient: −0.281, P < 0.001), primarily involved in anti-inflammatory responses and tumor promotion. These findings underscore the association between high TIL scores and enhanced anti-tumor immune responses, which could suggest a protective role of high TIL scores against metastasis.

**Fig. 7:**
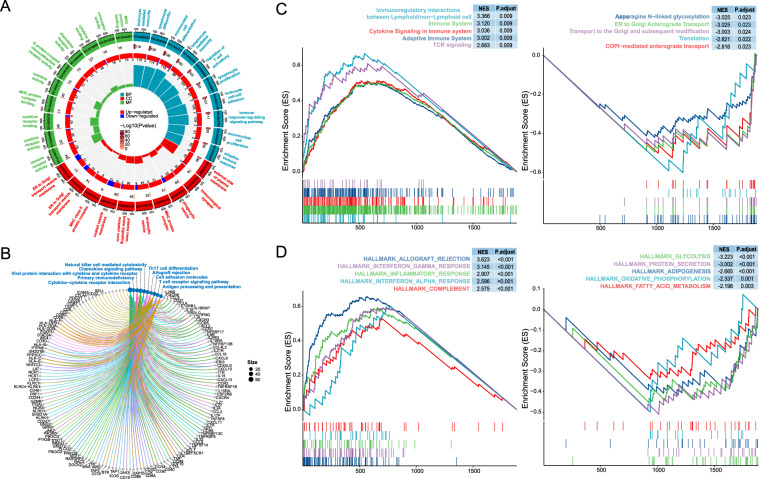
Biological relevance of TIL scores. **A** GO analysis. The circular plot consists of four concentric rings. The outermost ring represents the top 10 enriched GO terms from three different functional categories (biological process, BP, cellular component, CC, molecular function, MF). The scale on the outer ring indicates the number of genes within each GO term, and the colored blocks are labeled with the GO term IDs. In the second ring, moving inward, the length of each rectangle represents the number of genes enriched in each GO term (for example, in the top 1000 genes most correlated with TIL scores, the GO:0002443 term contains 118 enriched genes), with the length proportional to the scale in the outer ring. The color of the rectangles indicates the enrichment p-value of each term, with darker colors representing smaller p-values. The third ring shows the number of up-regulated and down-regulated genes for each term (the total number of upregulated and downregulated genes for each term corresponds to the gene count in the second ring). The innermost ring displays the enrichment score bars. The height of the bars represents the proportion of the gene count in the second ring to the total number of genes for the respective GO term. The scale of bars is set from 0 to 0.15. **B** The cnet plot of KEGG analysis. The top 10 enriched KEGG pathways are shown. The value of size represents the number of genes enriched under each pathway. **C**-**D** gene set enrichment analysis (GSEA). Figure 7 C shows the top five up-regulated (left) and down-regulated (right) Reactome pathways. Figure 7D shows the top five up-regulated (left) and down-regulated (right) Hallmark pathways. NES, normalized enrichment score

The top 30 GO terms from biological processes (BP), cellular components (CC), and molecular functions (MF) ontologies and the top 10 pathways related to increased TIL scores are presented in Figs. 7A and B, respectively. Notably, all enriched BP terms and KEGG pathways were immune-related. Furthermore, we employed gene set enrichment analysis (GSEA) to derive the top five upregulated or downregulated Reactome and Hallmark pathways, respectively, associated with increased TIL scores (Fig. 7C–D). Upregulated pathways covered different aspects of the immune system, from interactions between immune cells to specific cell signaling pathways and the regulation of humoral and cellular immune responses. This suggests that tumors with high TIL scores are embedded in an active immune microenvironment. Downregulated pathways were involved in critical biological processes related to the synthesis, modification, and transportation of proteins and fundamental aspects of cell metabolism and function. This dual pattern suggests that high TIL score cases may experience a constrained metabolic state of tumor cells, possibly due to immune-mediated stress in the tumor microenvironment.

## Discussion

In this study we investigated the links between TIL features derived from WSIs and LNM in LUAD. By integrating the TIL features (TIL scores and spatial clusters) with clinical risk factors, we observed a significant improvement in prediction performance, with AUC increases from 0.63, 0.61, and 0.69 (clinical model) to 0.78, 0.72, and 0.80 (integrated model) on the training, validation, and test sets, respectively. Patients with high TIL scores and/or classified as sTILC2 were associated with a reduced risk of LNM, which was confirmed on the independent test set. Incorporation of TIL scores led to a change in the predicted absolute LNM probability by an average of 5.06 and 4.06 percentage points in the training and validation cohorts, and by 6.32 percentage points in the test cohort. To our knowledge, this is the first study to assess the influence of TIL characteristics on LNM in LUAD.

The status of LNM is a significant prognostic indicator and is critical throughout the entire NSCLC patient management process, especially for those patients deemed suitable for curative therapy [5]. The assessment and management of LNs is therefore essential for determining the most appropriate therapeutic approach. However, in patients with early-stage NSCLC who were node-negative according to presection sampling, complete mediastinal lymph node dissection did not improve survival, as evidenced by the randomized phase 3 trial, AcosoG Z0030 [36, 37]. Therefore, identifying risk factors associated with LNM aids in recognizing high-risk groups and individualized treatment strategies. In this context, some studies have investigated risk factors, including lymphovascular invasion, degree of differentiation, and tumor size, which are commonly reported in the literature [38–40]. Furthermore, tumor size and age have been identified as independent risk factors in a large population study [17].

Recent evidence highlights a significant correlation between TIL density and improved prognosis in LUAD in the context of immunotherapy [13, 15, 41]. Studies have demonstrated an association between high TIL density and LNM in various cancers including early gastric cancer [42], breast cancer [43], melanoma [44–46], lip and oral cavity squamous cell carcinoma [47]. Generally, a high density of TILs appears to be a protective factor against LNM.

In LUAD specifically, it has been reported that CD8 + tumor-infiltrating cells exhibit more pronounced dysfunction and are found within a more immunosuppressive microenvironment in patients with LNM compared to those without LNM [48]. This suggests that the status of TILs may be closely linked to the presence of LNM. However, the association between the density and spatial characteristics of TILs with LNM has so far remained unknown. We have previously developed a pan-cancer approach, TILScout (20), which automates the calculation of TIL scores and the creation of global TIL maps from WSIs, significantly enhancing the efficiency of TIL assessment. Furthermore, we utilized deep learning techniques to extract features from global TIL maps and conducted clustering analyses to categorize the spatial distribution of TIL clusters across various samples. We defined and delineated two types of TIL clusters—sTILC1 and sTILC2. Our findings reveal that both TIL scores and spatial clusters correlate with LNM and contribute independent and complementary information to the T stage in predicting LNM risk. Notably, TIL scores exhibited a particularly strong association, which we validated in an independent test set. TIL features may serve as a histology-based adjunct for risk stratification, especially when nodal evaluation is incomplete or inconclusive, offering complementary value beyond clinical and radiological assessments. Nevertheless, integration of such computational tools into clinical pathology workflows will require several practical steps. These include the availability of digital slide infrastructure (for example, whole-slide scanners and viewers) and, more importantly, prospective clinical validation to confirm clinical benefit and usability. Addressing these translational challenges will be essential for the future clinical adoption of TIL-based biomarkers.

Our study has several limitations that should be noted. First, statistically significant differences in age, T stage, and TIL scores between the TCGA and XY cohorts could reflect institutional heterogeneity, which potentially impacts the generalizability of our findings despite the robust predictive performance in the independent XY cohort. Future validation with larger, prospective, and multi-institutional cohorts encompassing broader clinical diversity is therefore essential. Second, the limited number of cases within specific subgroups and the lack of information on driver gene mutations in our datasets prevented reliable subgroup analyses based on critical molecular markers such as EGFR or KRAS mutations, or different clinical stages. Given that genetic alterations may influence tumor immunity and metastatic behavior[49], future studies integrating genomic data with spatial TIL features will help clarify how tumor-intrinsic factors interact with immune infiltration to affect LNM. Finally, although our GSEA results provided biological context of the association between high TIL scores and constrained metabolic state of tumor cells, these findings remain statistical correlations rather than direct evidence of causality. Further mechanistic investigations using spatial transcriptomics or functional validation methods are warranted to elucidate the precise role of TILs in metastasis. Addressing these limitations and future directions will strengthen the biological interpretability of TIL-based biomarkers and promote their translational potential for clinical pathology workflows.

In conclusion, spatial features of TILs in primary tumor tissues are associated with LNM in LUAD. Patients with low TIL scores and/or classified as the sTILC1 type are associated with an increased risk of LNM. These findings potentially contribute to recognizing high-risk groups and developing personalized treatment strategies.

## Supplementary Information

Additional file1

## Data Availability

TCGA-LUAD WSIs, clinical, and transcriptomics data can be downloaded via the GDC (https://portal.gdc.cancer.gov/). The independent test data (XY cohort) that support the findings of this study are available from the corresponding author upon reasonable request. TILScout is available via the GitHub repository: https://github.com/huibozh/TILScout. The codes regarding hotspot analysis and sTILCs are available via the GitHub repository: https://github.com/huibozh/sTILCs.

## Abbreviations

LNM: Lymph node metastasis
LUAD: Lung adenocarcinoma
TILs: Tumor‐infiltrating lymphocytes
sTILCs: Spatial TIL clusters
NSCLC: Non-small cell lung cancer
WSIs: Whole slide images
TCGA: The Cancer Genome Atlas
PCA: Principal Component Analysis
GO: Gene Ontology
KEGG: Kyoto Encyclopedia of Genes and Genomes
GSEA: Gene Set Enrichment Analysis
NES: Normalized enrichment score
ROC: Receiver operating characteristic
AUC: Area under the ROC curve
SHAP: SHapley Additive exPlanations
DCA: Decision curve analysis
NMRFMs: Node Metastasis Random Forest Models
PCCs: Pearson correlation coefficients
