# Supplementary material for: Spatial features of tumor-infiltrating lymphocytes in primary lesions of lung adenocarcinoma predict lymph node metastasis
Source: J Transl Med. 2025 Jul 25;23:842. doi: 10.1186/s12967-025-06860-1 (PMC12297629; doi:10.1186/s12967-025-06860-1)
Supplement: Supplementary file 1 — Additional file1 [file 12967_2025_6860_MOESM1_ESM.pdf]

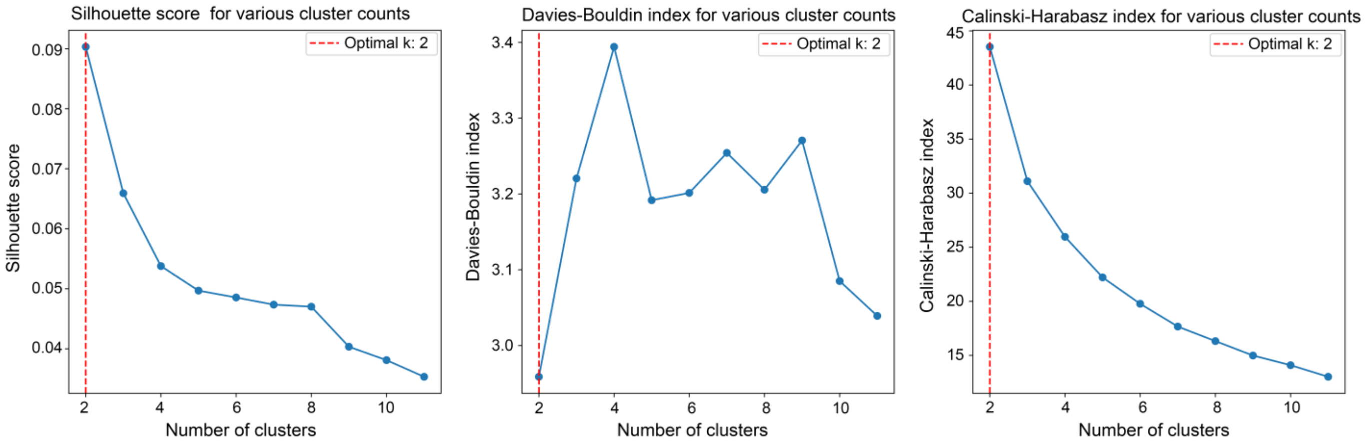

Supplementary Figure 1. The optimal number of clusters determined based on the Silhouette Score, Davies-Bouldin Index, and Calinski-Harabasz Index.

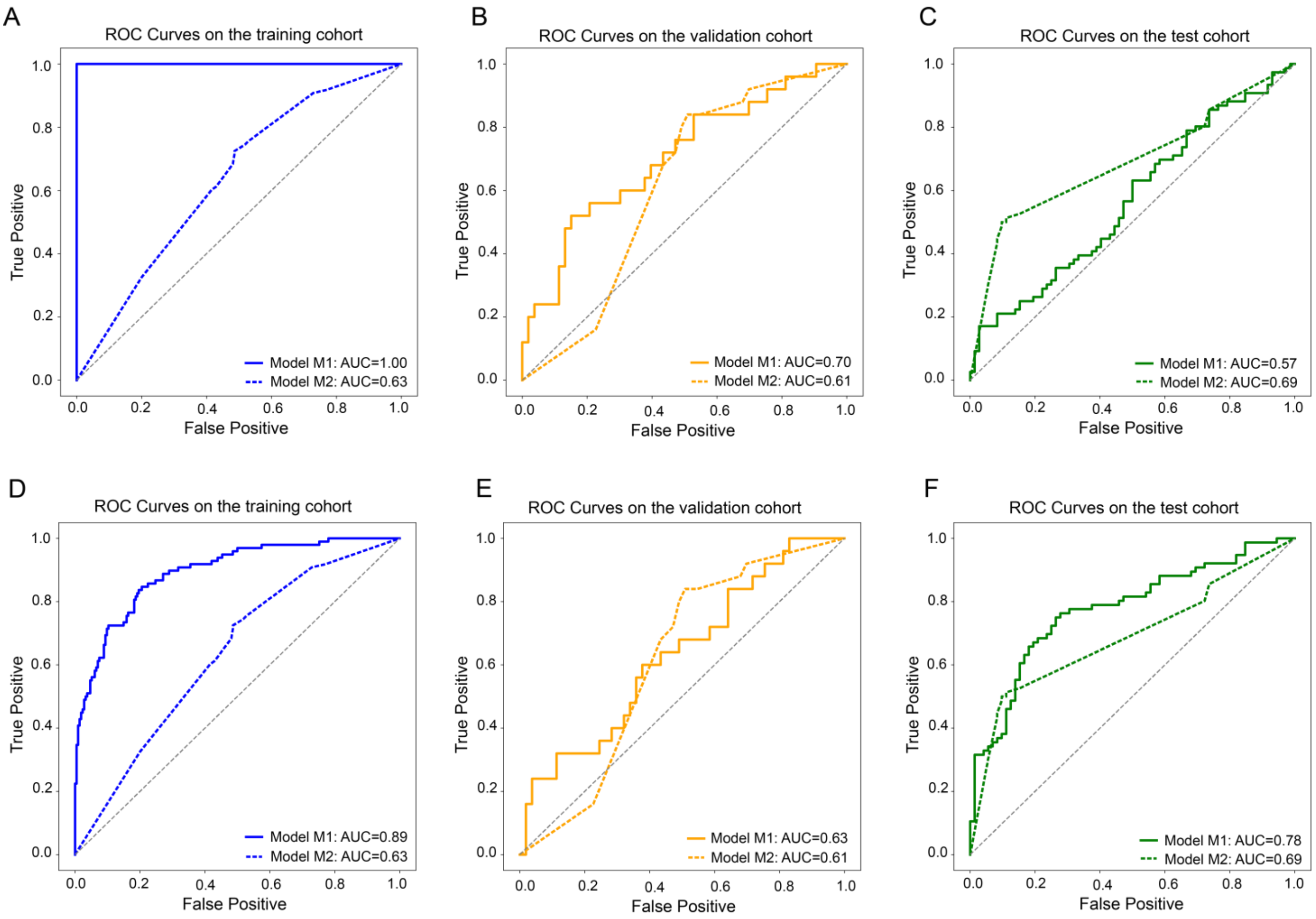

Supplementary Figure 2. The performance of model M1 on the training, validation, and test cohorts under two additional scenarios. A-C, model M1 performance under the first scenario (including all 244 PCA-derived features in place of the sTILCs). D-F, model M1 performance under the second scenario (using only the two most important principal components (PC1 and PC2) instead of the sTILCs).
